# Case Report:clinical experience of bilateral giant pediatric Testicular adrenal rest tumors with 3 Beta-Hydroxysteroid Dehydrogenase-2 family history

**DOI:** 10.1186/s12887-021-02883-x

**Published:** 2021-09-15

**Authors:** Lingyun Yu, Pengyu Chen, Wenbin Zhu, Junjie Sun, Shoulin Li

**Affiliations:** Department of Urology, Shenzhen Children’s Hospital, Shenzhen, Guangdong China

**Keywords:** TARTs, Testicular adrenal rest tumors, congenital adrenal hyperplasia, 3β-hydroxysteroid dehydrogenase deficiency-2, case report

## Abstract

**Background:**

We reported a patient with Testicular adrenal rest tumors(TARTs) caused by congenital adrenal hyperplasia(CAH). TARTs occur frequently in CAH population with 21-hydroxylase deficiency(21-OHD). There are few reports of TARTs with 3β-hydroxysteroid dehydrogenase deficiency-2 (3β-2HSD).Furthermore,gaint TARTs are rarely mentioned in reported cases involving affected siblings.

**Case presentation:**

A 14-year-old male patient was admitted by congenital adrenal hyperplasia with progressively increasing bilateral testicular masses.The Patient and his elder brother had been performed mutational and chromosome analysis and biopsy. Hormonal and anthropometric measurements were performed during endocrine treatments. We successfully performed surgery and excised two 83mm×46mm×44mm and 74mm×49mm×31mm tumors. Our pathology and immunochemistry tests have proven TARTs in patient. At first, both siblings received regular doses of hydrocortisone and fludrocortisones and tumor size regressed. During the one-year irregular intake due to Covid-19 pandemic, endocrine treatment became insensitive and tumor size slowly increased. The gene analysis reported two novel mutations C.776 C>T and C.674 T>A. The C.776 C>T is from father and has been reported. The C.674 T>A inherited from mother and cannot found in gene library and may related to TARTs.

**Conclusions:**

This case illustrates inadequate hormone therapy could cause tumor enlargement. It is essential to seek for ultrasound examination once suspected scrotal mass occurred.It is necessary to adjust endocrine medicine or adopt surgery in refractory gaint TARTs. And presence of tunica vaginalis cavity may indicate the severity of TARTs in surgery.

**Supplementary Information:**

The online version contains supplementary material available at 10.1186/s12887-021-02883-x.

## Background

CAH is defined as autosomal recessive disease group mainly caused by enzymes defects that block adrenocorticosteroid synthesis-metabolism and cause disorders of sex development(DSD),which are common in female births and the reported incidence is about 1: 15,000[1].CAH patients are always categorized as salt-wasting(SW),simple-virilizing(SV),non-classic(NC) types and it is essential for them,especially for SW type,to adopt life-long exogenous hormone therapy due to glucocorticoid (or with mineralocorticoid) deficiency and increased androgen secretion.Currently, several key synthesis enzyme genes were recognized as mutation sites and CAH were classified as 21-OHD,11β-hydroxylase deficiency(11β-OHD),17α-hydroxylase deficiency(17α-OHD),3β-2HSD and lipoid congenital adrenal hyperplasia(LCAH).The manifestations,mostly seen in 21-OHD, were suprarenal gland crisis and water-electrolyte imbalance.However,testicular adrenal rest tumors(TARTs),originated from testicular ectopic adrenal tissue, were occasionally reported in and correlated with lower fertility and infertility in male CAH group.In general,TARTs were always mentioned in 21-OHD and 11β-OHD.We reported one child with TARTs led by 3β-2HSD with family medical history after failure of endocrinology treatment and has accepted surgery in our hospital.

## Case presentation

**Fig. 1:**
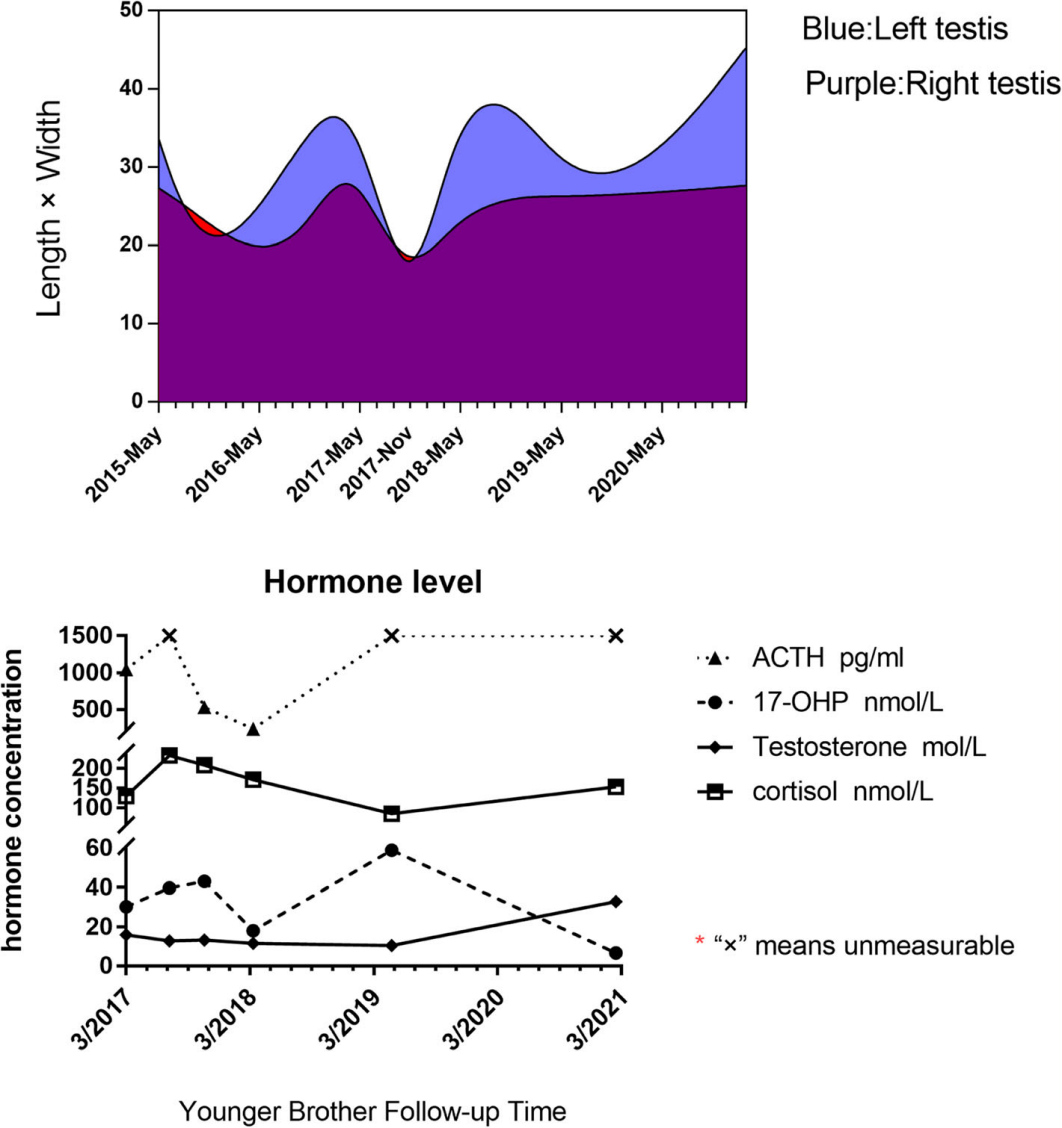
We drew line graph(see in Fig. 1) to illustrate TARTs changes by incorporating various harmones.It is noteworthy,describled by parents,that both of siblings showed hypospadia after birth and the whole body skin became dark in 3 years old

A 14-year-old boy was admitted for bilateral scrotal masses for 11 years in our outpatient department. According to the previous documents provided by his family, his parents were normal but his elder brother showed same scrotal masses.When they were 3 years old,the elder brother,who is 10 years older than the younger, accepted serious hypospadias repair surgery in 1999 while the younger in 2009 at Wuhan Children’s Hosptial. In the short postoperative period, this younger child’s skin color became tanned and the color deepen. Although parents noticed that his testicles were slightly larger than peers in progressive pigmented stage, occasionally even with orchialgia, same to childhood of his elder brother, they still ignore it and did not take him to medical treatment until oversize bilateral lumps 5 years later. Thus his parents sent them to Tongji Hosiptal affiliated to Middle China University in Wuhan, and both were diagnosed as TARTs with 3β-2HSD by routine hormone examination(see in Fig. 1) and recommended gene analysis(see in Supplementary Fig. 1).In addition, tumor tissue biopsy approved by custodians also supported TARTs diagnosis. The markedly elevated androgen, ACTH and 17-OHP levels and actual bone age(15-year-old) indicated only hydrocortisone(HC) supplementary therapy feasible when they refused surgerical excision. In long-term follow-up, HC dosage(15 mg/m^2^·d) was gradually adjusted to 23 mg/m^2^·d, which was proven effective by diminshed tumor size. While two siblings often forgot to take medicine on time for one year and did not seek for regular examination due to COVID-19 epidemic in Wuhan. The bilateral TARTs size became slightly larger than pre-treatment and seemed insensitive to HC.

**Fig. 2:**
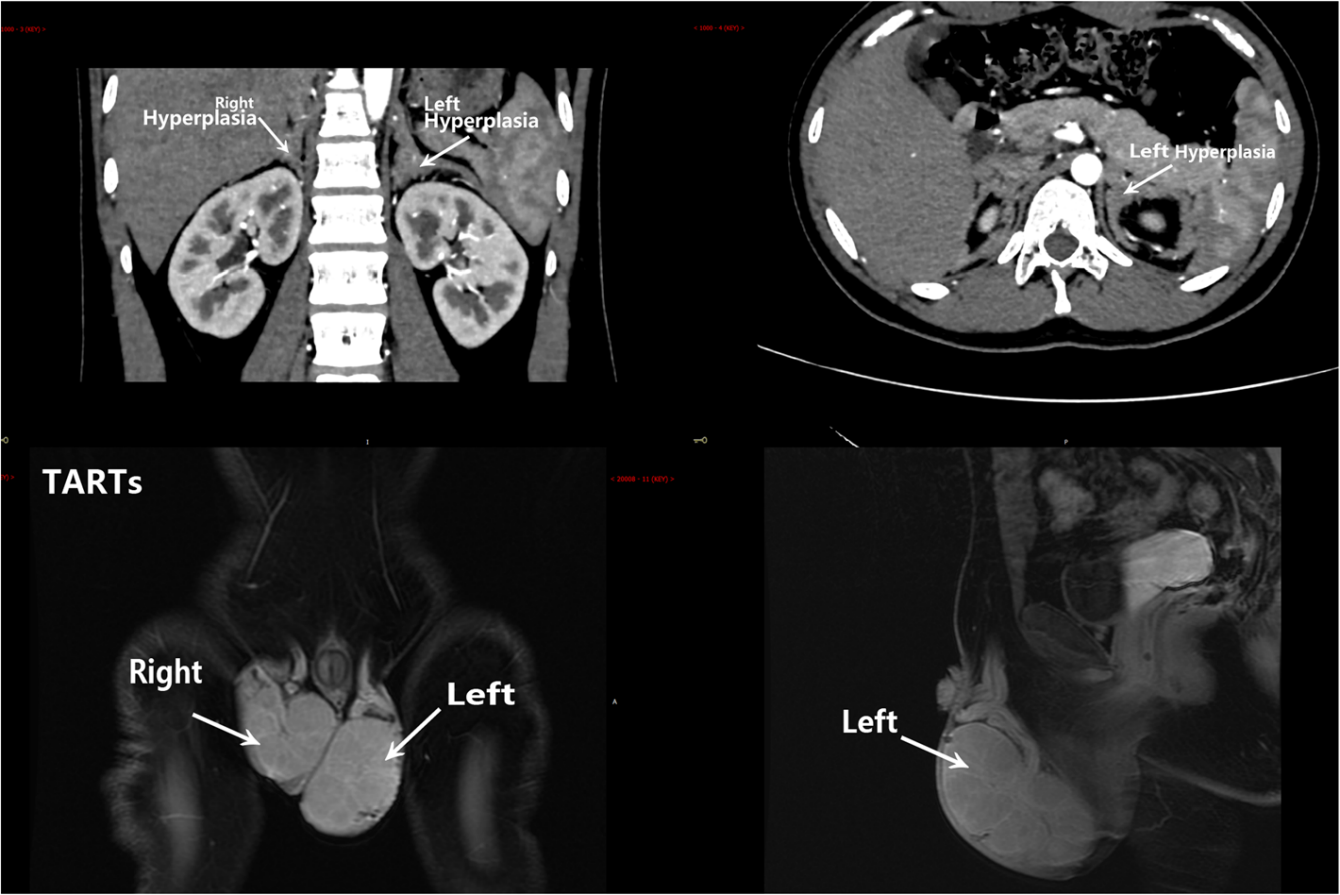
Bilateral adrenal hyperplasia showed in enhanced computed tomography(CT).The internal and external branches and junctions of bilateral adrenal glands were diffusely thickened, and the thickest part was about 20 mm.Oval masses,found in bilateral scrotum, were about 65mm × 51mm × 65mm(Left), 53mm × 58mm × 54mm(Right) (AP××LR ×UD ) The signal was less uniform, mainly slightly high signal on T1WI and T2WI (compared with muscle), with more strip shaped low signal septa on T1WI and T2WI, high signal on DWI and no obvious bleeding signal

In the second admission to Shenzhen Children’s Hospital, we conducted sonography and computed tomography(CT)(show in Fig. 2).The Physical examinations details included:Height_145.4 cm(-3.4 SD), Sitting Height_84.6 cm, Weight_55.7 kg, Pigmented Skin(see Supplementary Fig. 2). Tanner staging: breast B1, armpit hair A2,pubis PH5,penis 6 cm*2.7 cm.

With slowly increased height about 1.2 cm since first admission 5 years ago, we replaced HC with dexamethasone(DXMS) to control the disease. The CT image suggested that testis tumor nearly replaced normal testicles tissue and hyperplasia in bilateral adrenal cortex. After Multi-disciplinary discussion, the tumor was proposed to excise to avoid growing up and his parents accepted our advice.

**Fig. 3:**
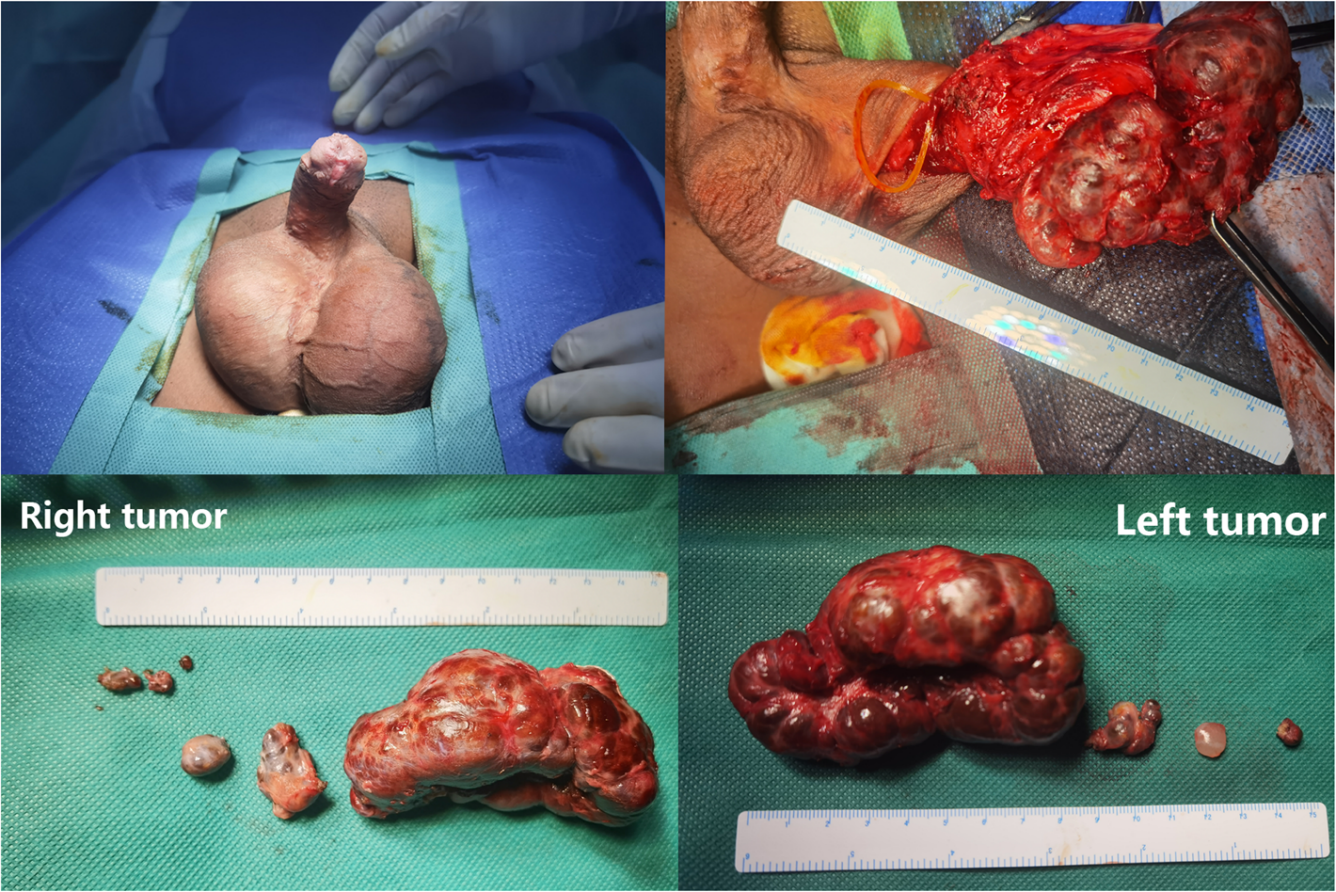
The excised tumors(Left,Right).Left: 83mm×46mm×44 m; Right: 74mm×49mm×31mm

After the incision into tunica vaginalis in each side, there is an obvious characteristic that cavity of tunica vaginalis disappeared, we can noticed two hard giant tumors(see Fig. 3) with several sporadic bean-like tubercles and cannot clearly distinguished structure of epididymis and testis. Finally,we stripped the tumor and suture incision by rolling residual testicular tissue and tunicae vaginalis.The tumors were demonstrated TARTs by pathology results (see in Supplementary Fig. 3 and Table 1 ) with no existence of Reinke crystals.

In the postoperative week,bilateral scrotum skin folds gradually resumed with subsidence of scrotum swelling and bilateral size obviously decreased comparing with primary manifestation.

During the third admission to Shenzhen Children’s Hospital in 2021 July, physical examinations included:Height_146.2 cm(-3 SD), Sitting Height_85.3 cm, Weight_64.5 kg, Pigmented Skin color improved. Tanner staging: breast B1,armpit hair A2,pubis hair PH5,penis 1 cm*3.5 cm.The surgery incision recovered well and bilateral testis unltrasound show Left volume was 11.59ml and Right was 10.79ml. Adrenal hormone profile shows ACTH 4.090pg/ml, testosterone 7.54nmol/L,17-OHP 0.47nmol/L, serum cortisol is 0.000ug/dl and follow-up is necessary for long-term prognosis.

## Discussion and conclusions

3β-2HSD is a rare type of congenital adrenocortical hyperplasia, accounting for about 5 % of CAH.In 3β-2HSD patients,SW type is usually confirmed in the neonatal period whereas the non-SW type before puberty[2].3β-HSD deficiency is caused by the deficiency of 3β-HSD2 and peripheral 3β-HSD1 is normal.During the fetal period, the activity of 3β-HSD2 remain high level to promote external genitalia development before 12 weeks in pregnancy[3].Therefore,the lack of 3β-HSD2, in early stage of fetus,can damage the biosynthesis of testosterone at testis, resulting in incomplete masculinization, even complete feminization of external genitals.However, peripheral 3β-HSD1 activity peaked after the development of external genitalia (that is, the middle or later stage of pregnancy)and fail to develop normal male external genitalia,though 3β-HSD1 can mediate the conversion of excessive dehydroepiandrosterone to testosterone. For female group, lack of testosterone in early pregnancy leads to Wolffian duct degeneration and Müllerian duct evolve to fallopian tube and uterus.Be contray to middle or later stage in male pregnancy, testosterone will increase androgen level,through mediation to dehydroepiandrosterone by peripheral 3β-HSD1, and impose mild masculinization on female external genitalia,like clitoris enlargement and /or without labial fusion.

The TARTs was firstly described by Wilkins in 1940[4],and the prevalence was 25 % in childhood [5].Non-typical sites like pelvis, retroperitoneum, kidney, spinal cord or Non-CAH diseases have been proven existence of adrenal rest tumors other than testis and CAH[5, 6].At present,the etiology of TARTs was considered that chronic adrenocorticotropic hormone (ACTH) stimulates aberrant adrenocortical cells in testicular parenchyma.Most over 2 cm TARTs were detected by palpation while less 2 cm tumors were found in ultrasound and some TARTs regressed in the first year of life [6, 7]. In fact,most TARTs location is in the middle site of testis[7, 8]that is undetectable in physical examination and that is why we recommend ultrasound as the first means to screen testis tumor. Most TARTs are hypoechoic and bilateral while vascularity remains controversial for lack of objective values,machine settings difference and subjective judgment[9, 10]. In Magnetic Resonance Imaging(MRI), TARTs characterized with hypointensity on T2-W images, and hyperintensity or iso-intensity on T1-W images and lesion margins were clearly defined[5].Owing to overlapping image results in Leydig tumors, clinical information and laboratory testing should be combined correct diagnosis.In fact, Miroslav Dumic[11] found that TARTs patients have been in stage 3 or 4 which should have accepted the early ultrasound examination. Since long-term over-exposure to ACTH will promote tumor growth in stage 2 and proceed into next stage, which is expected that tumor growth will restart after decreasing the dose of glucocorticoids in stage 3[12].The RECIST( Response Evaluation Criteria in Solid Tumors ) has proven previous tumor treatment ineffective in this child[13]. Therefore we excuted the surgery for this patient.

The TARTs origin recently was considered that it was related to a more pluripotent steroidogenic cell type that is already present in utero, probably originating from the adrenogonadal primordium or from the urogenital ridge[5, 12].In addition,the excessive growth of tumor was hypothesized that may related to the ACTH/ Angiotensin II(Ang II) [14] or luteinizing hormone(LH)[12].GATA transcription factor may also promote TARTs evolution[15].While an inadequate hormonal control can led to a 16 times greater chance of 21-OHD CAH patient to present with TARTs and hormonal treatment reduction may induce renewed growth[5].Hormonal control was defined as inadequate when 50 % or more of serum androstenedione results were above reference values, and 50 % or more of serum 17-OHP concentrations were over 5 times the upper limit of reference values per age[16].Thus the 3β-2HSD CAH patient should follow regular and low-risk hormonal therapy dosage,and overdose may cause tumor evolution and lower expected height[7, 16].However,there is no enough data to estimate rational choice of harmone like dexamethasone.It is necessary for attempt of increasing Gamma Knife Surgery (GKS) doses to avoid excess endocrine treatment and estimate if the growth of the tumor is reversible in the I to III phase[17, 18].It is essential that we should calculate the prevalence of TARTs without medicine intervention,especially failure of hormonal treatment.

Although Claahsen-van[17] has described the TARTs evolution,Stage 5 characteristics of the classification is not clear.In this case,the mentioned diappearing cavity of tunica vaginalis,which may caused by compression of tumor growth,can be used to explicitly describe TARTs satges for determining surgical treatment. Tunica vaginalis[19] is a mesothelium-lined sac that results from closure of the superior portion of the processus vaginalis, which is thought to be mediated by androgen, smooth muscle cell[20] and autonomic nervous system[21]. In spite of unclear mechanism preventing tunica vaginalis obliterated, Hutson[22] found tunica vaginalis cavity volume shrinks with the age in postmortem adult groups.It is necessary to explore the potential impact on tunica vaginalis in endocrine diseases population accompanied by ubiquitous hormone disorder.

Besides,fertility loss is deemed as the complication in TARTs cases and always related mechanical obstruction and toxic effect of local adrenal steroids or metabolites derived from adrenal rest tumors[23].In spite of infertility,some effective treatments have been proposed like follicle-stimulating hormone(FSH)/LH induction or mitotane therapy[24].In most azoospermia or oligozoospermia patients affected by CAH,there are several reversible fertility TARTs cases[25],which could provide some guidance for 3β-2HSD population.

The cavity of tunica vaginalis may indicate the severity of TARTs.It is essential to seek for ultrasound examination once suspected scrotal mass occurred. It is necessary to adjust endocrine medicine or adopt surgery when regular and adequate hormone therapy was ineffective or refractory gaint TARTs emerged.Semen analysis were considered an assistant means to preserve fertility in male CAH patients.

## Supplementary information

Additional file 1 Supplementary Fig. 1: Gene analysis showed there were two mutations in siblings’s chrosome 1. c.674T > A occurs at chr1:119,964,900 and c.776 C > T occurs at chr1:119,964,798.

Additional file 2 Supplementary Fig. 2: The pigmented skin in lower limbs and abdomen. Supplementary Fig. 3: (A) Fibrous tissues separate tumor into multi-nodular masses.(B)(C)(D)Images display several molecule results in IHC staining.Method: Olympus BX53 and Axiocam 305 color were used to capture the microscopy images, the horizontal and vertical dpi are 300 in 4 images. We add scale bar to these images by Zen 2.6 (blue edition) without enhancement and merge them into

Additional file 3 Supplementary Fig. 3 by Adobe Illustrator CC 2017.

Additional file 4 Supplementary table: Table 1–1 and Tables 1 and 2 presents related molecule for indentification in TARTs.

## Data Availability

The datasets used and/or analysed during the current study available from the corresponding author on reasonable request.

## Abbreviations

TARTs: Testicular adrenal rest tumors
CAH: congenital adrenal hyperplasia
3β-2HSD: 3β-hydroxysteroid dehydrogenase deficiency-2
DSD: disorders of sex development
SW: salt-wasting
SV: simple-virilizing
NC: non-classic
21-OHD: 21-hydroxylase deficiency
11β-OHD: 11β-hydroxylase deficiency
17α-OHD: 17α-hydroxylase deficiency
3β-2HSD: 3β-hydroxysteroid dehydrogenase type II
LCAH: lipoid congenital adrenal hyperplasia 12
HC: hydrocortisone
CT: computed tomography
MRI: Magnetic Resonance Imaging
ACTH: adrenocorticotropic hormone
DXMS: dexamethasone
SD: Standard Deviation
RECIST: Response Evaluation Criteria in Solid Tumors
Ang II: Angiotensin II
FSH: follicle-stimulating hormone
LH: luteinizing hormone
GKS: Gamma Knife Surgery
