# Supplementary figures and images for: Case Report:clinical experience of bilateral giant pediatric Testicular adrenal rest tumors with 3 Beta-Hydroxysteroid Dehydrogenase-2 family history
Source: BMC Pediatr. 2021 Sep 15;21:405. doi: 10.1186/s12887-021-02883-x (PMC8440148; doi:10.1186/s12887-021-02883-x)

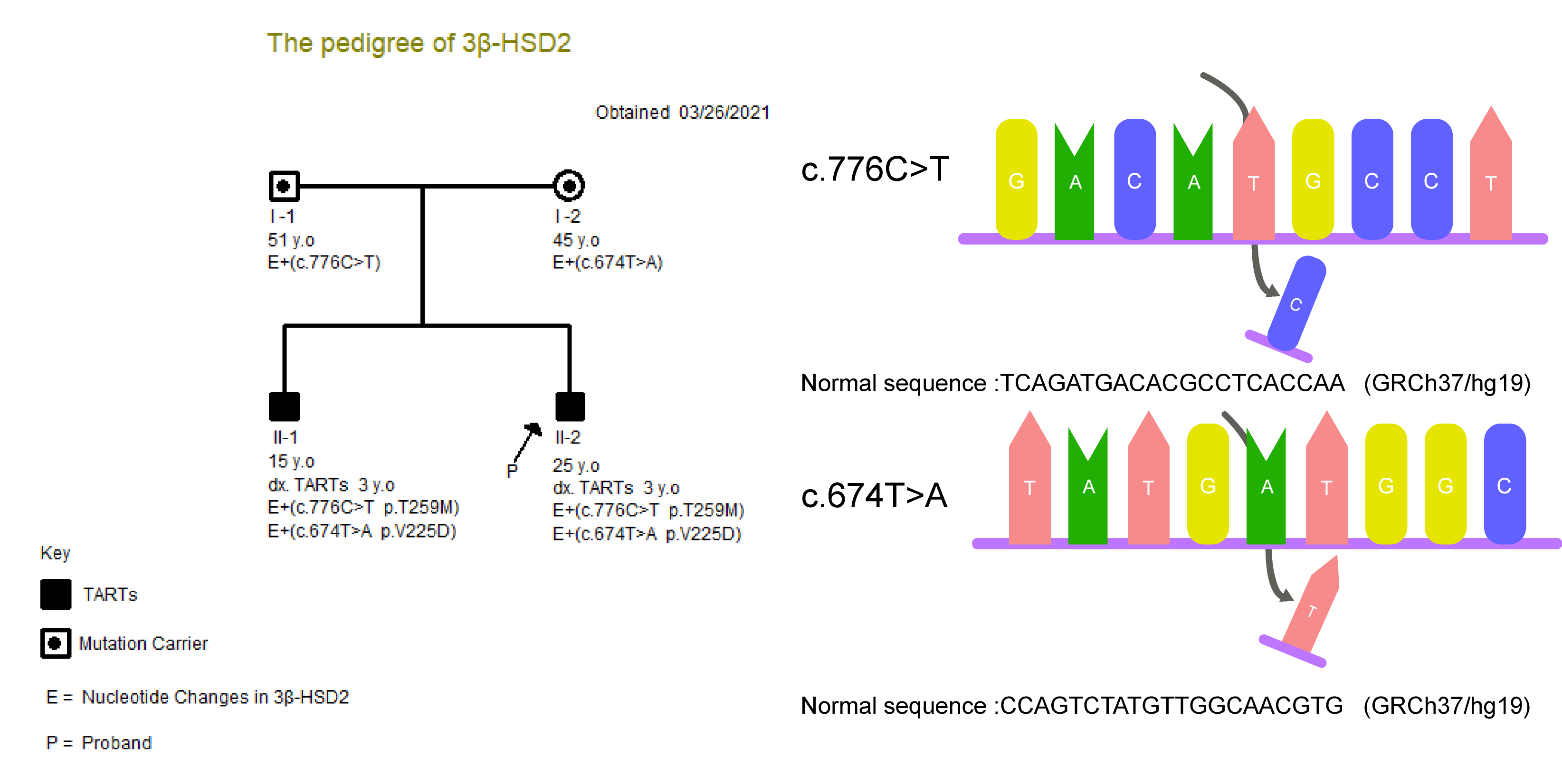

Supplement: Supplementary file 1 — Supplementary Fig. 1: Gene analysis showed there were two mutations in siblings’s chrosome 1. c.674T > A occurs at chr1:119,964,900 and c.776 C > T occurs at chr1:119,964,798. [file 12887_2021_2883_MOESM1_ESM.tif]

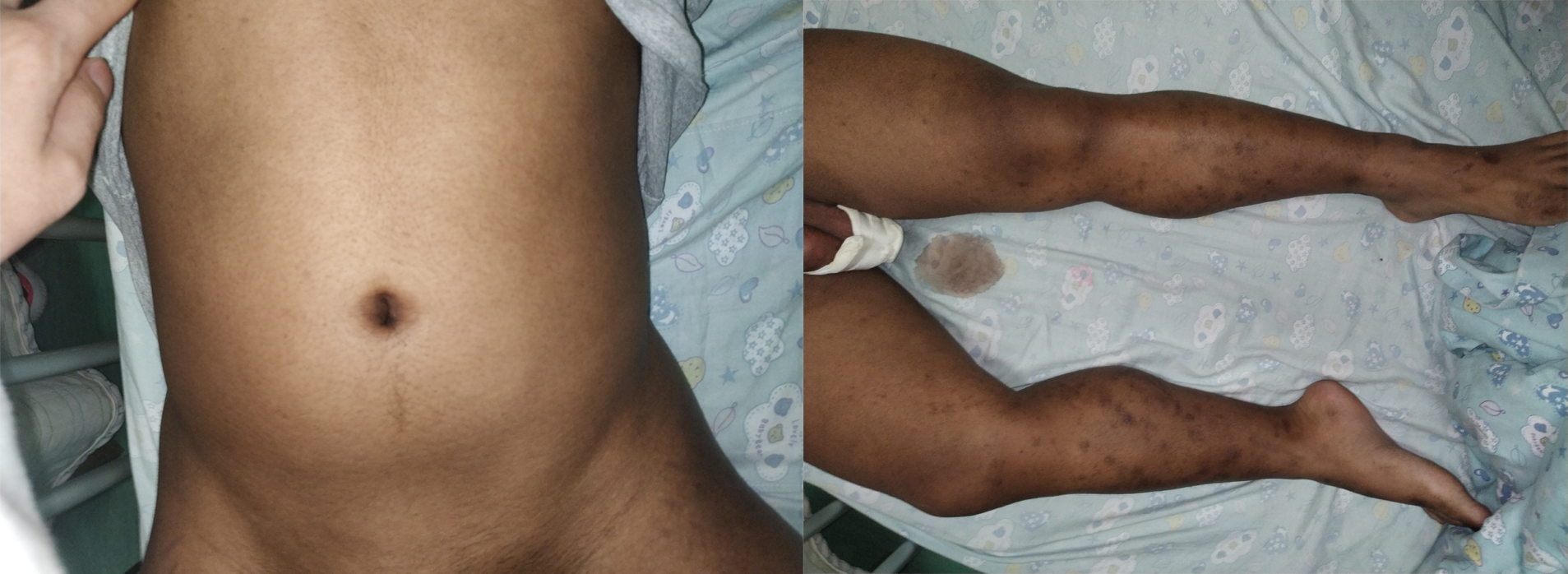

Supplement: Supplementary file 2 — Supplementary Fig. 2: The pigmented skin in lower limbs and abdomen. Supplementary Fig. 3: (A) Fibrous tissues separate tumor into multi-nodular masses.(B)(C)(D)Images display several molecule results in IHC staining.Method: Olympus BX53 and Axiocam 305 color were used to capture the microscopy images, the horizontal and vertical dpi are 300 in 4 images. We add scale bar to these images by Zen 2.6 (blue edition) without enhancement and merge them into [file 12887_2021_2883_MOESM2_ESM.tif]

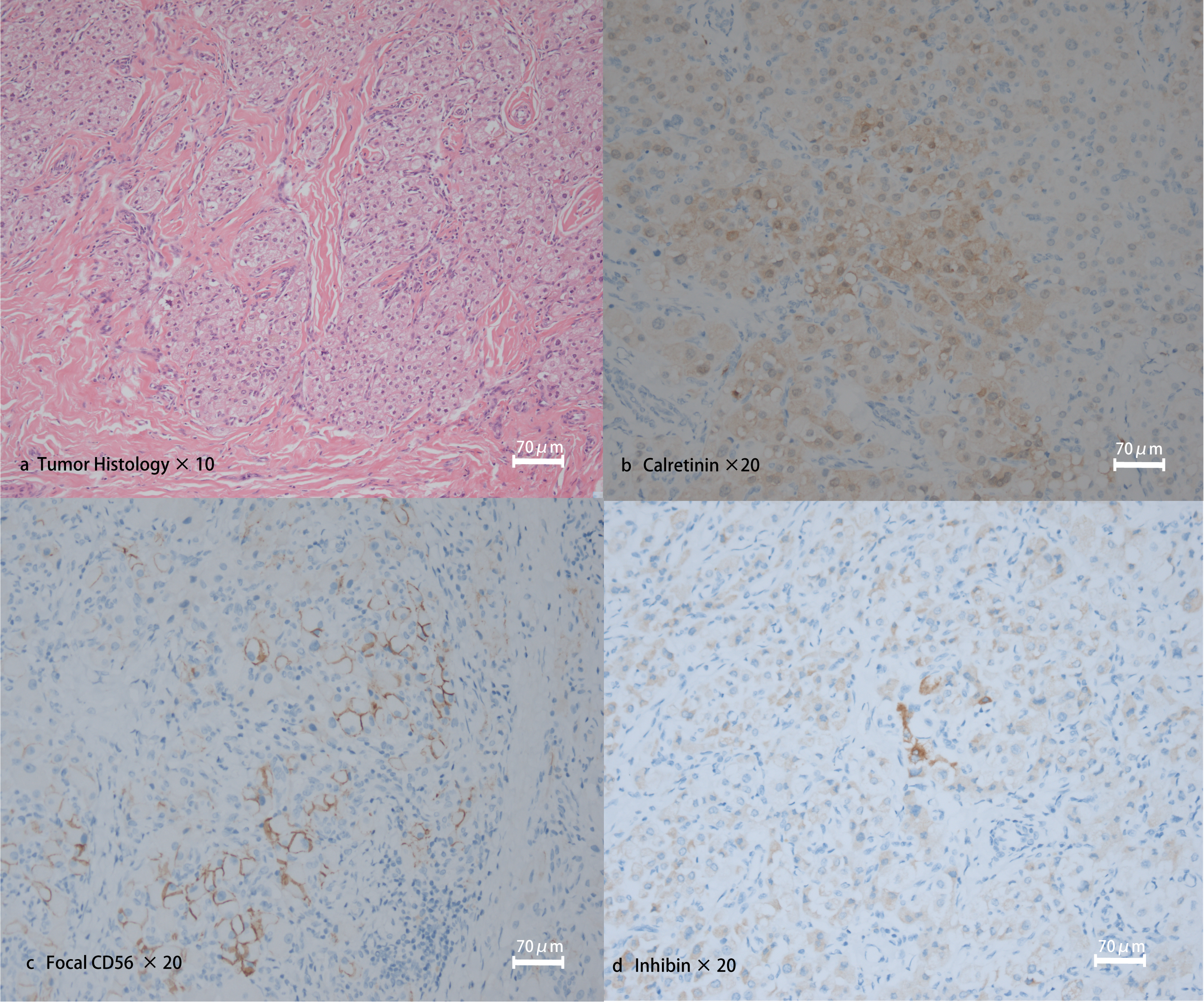

Supplement: Supplementary file 3 — Supplementary Fig. 3 by Adobe Illustrator CC 2017. [file 12887_2021_2883_MOESM3_ESM.tif]
