# Supplementary material for: Case Report:clinical experience of bilateral giant pediatric Testicular adrenal rest tumors with 3 Beta-Hydroxysteroid Dehydrogenase-2 family history
Source: BMC Pediatr. 2021 Sep 15;21:405. doi: 10.1186/s12887-021-02883-x (PMC8440148; doi:10.1186/s12887-021-02883-x)
Supplement: Supplementary file 4 — Supplementary table: Table 1–1 and Tables 1 and 2 presents related molecule for indentification in TARTs. [file 12887_2021_2883_MOESM4_ESM.docx]

**Table 1-1**

| Tumor IHC | CD99 | CgA | Calretintin | Inhibin-α | Melan A | PHOX-2B | Syn | Ki-67 | TSPY | CD56 |
| --- | --- | --- | --- | --- | --- | --- | --- | --- | --- | --- |
| Right | — | — | Partially weakly positive | Partially weakly positive | — | — | Less weakly positive | 3% | / | Focal  weakly positive |
| Left | / | — | / | / | / | — | Less sporadic  positive | 1% | +/- | Less sporadic  positive |

**Table 1-2**

| Peri-tumor seminiferous tubule-like tissue specimen | PLAP | Oct3/4 | TSPY |
| --- | --- | --- | --- |
| Right | — | — | +/- |
| Left | — | — | +/- |
